# Psychological impact of COVID-19: Assessing the COVID-19-related anxiety, individual’s resilience and conspiracy beliefs on attitudes to COVID-19 vaccination

**DOI:** 10.3389/fpsyg.2022.906914

**Published:** 2022-08-11

**Authors:** Nadzirah Rosli, Elaina Rose Johar, Nursyafinaz Rosli, Nor Fazilah Abdul Hamid

**Affiliations:** ^1^Faculty of Economics and Management, Universiti Kebangsaan Malaysia, Bangi, Malaysia; ^2^Malacca Unified Command Centre, Melaka, Malaysia; ^3^Hospital Melaka, Melaka, Malaysia; ^4^Institute of Technology Management and Entrepreneurship, Universiti Teknikal Malaysia Melaka, Melaka, Malaysia

**Keywords:** anxiety, conspiracy beliefs, resilience, COVID-19, vaccination, attitudes

## Abstract

It has been 2 years since the first outbreak of the COVID-19 virus, and continuous efforts and measures have been exerted and implemented to halt its spread, such as the introduction of vaccination programs. However, as with the consumption of other products and services, some people hold different beliefs, consequently affecting their attitudes toward COVID-19 vaccination. Thus, vaccine unwillingness and hesitancy remain an enormous concern for many countries. This paper explores the effects of anxiety, individual resilience, and conspiracy beliefs on attitudes toward COVID-19 vaccines among the population of Malaysia—with a focus on Muslim individuals. We used survey data from 438 respondents (205 male, 233 female) to assess the research model. To conduct the multi-group analysis, we used partial least square structural equation modeling in SmartPLS 3. The results suggest that anxiety is positively associated with COVID-19 vaccination attitudes, whereas conspiracy beliefs have an inverse effect on vaccination attitudes, while an individual’s resilience is also positively associated with vaccination attitudes. Furthermore, it is found that the relationship between conspiracy beliefs and vaccination attitudes is weakened for an individual with a higher level of resilience. The findings also reveal the differences and similarities between males and females. To the best of our knowledge, this study is the first to simultaneously explore and demonstrate the effects of COVID-19-related anxiety, conspiracy beliefs and resilience with people’s attitudes toward COVID-19 vaccines and to examine the homogeneity of both males and females—especially among Malaysia’s Muslim population—thereby offering a valuable contribution to the literature.

## Introduction

Over the years, the world has gone through a number of pandemics, including SARS, H1N1, Ebola, Equine Flu, and more recently COVID-19. COVID-19 has caused harm to people in many ways, including the population’s mental health (Forte et al., 2020; Nguyen, 2021). It is evidenced that the psychological effects of infections and isolation measures are not only limited to the fear of getting the virus (Barbisch et al., 2015). Recent studies suggest that these measures have caused a wide range of psychological impact, that is significant and potentially prolonged, including psychological distress such as anxiety and suicidal thoughts (Pramukti et al., 2020), mood disorder and anxiety and post-traumatic stress disorder, sleep disorder, and several other psychological conditions (Brooks et al., 2020).

Several factors have caused the increase in mental health issues during the pandemic. According to some studies, the mental health issues are stemmed from economic concerns, while others believed that it is due to concerns over their health, and related to the social distancing measures that have been implemented (Kämpfen et al., 2020). The pandemics’ safety measures, along with the urge of returning to normalcy have directly and indirectly affected people’s social life and mental wellbeing. The interim measures, such as movement order control (MCO), physical distancing, and quarantine have shown to increase people’s fear, stress, and anxiety (Moni et al., 2021). Nevertheless, these precautionary measures were insufficient in halting the virus’s spread (Baeza-Rivera et al., 2021). Due to the positive impact on the control and prevention of infectious diseases in the past, the WHO issued a call for the development of vaccines in order to control the pandemic (Chan, 2017; Lurie et al., 2020). Similarly, recent studies suggest that the most effective way to combat COVID-19 is through vaccination (Ahorsu et al., 2022).

To limit the diffusion of the virus, the Malaysian government established a series of actions aimed at containing the spread of the epidemic. In Malaysia, the COVID-19 vaccination is provided voluntarily and provided free of charge to all Malaysian residents under Malaysia’s National COVID-19 Immunization Programme (PICK) (The Special Committee for Ensuring Access to Covid-19 Vaccine Supply [JKJAV], 2021). This means that people have been allowed to choose whether to get vaccinated or remain unvaccinated. Compulsory measures, on the other hand, are uncommon, and some degree of self-selection is preferred (Sasaki et al., 2022). As a result, there are people who are inclined to benefit from herd immunity acquired through others’ vaccination uptake while avoiding their vaccination. If a large number of people make this decision, society’s vaccination rate may fall short of herd immunity.

Vaccination has a direct (personal) benefit by eradicating or reducing the likelihood of infection with a specific disease. Furthermore, most vaccines indirectly benefit unvaccinated individuals by contributing to herd immunity (Pfattheicher et al., 2022). However, recently, more studies have discussed the health risk of not being vaccinated. In a study, the researcher found that unvaccinated individuals increased the risk of COVID-19 for everyone when they mix with a vaccinated individual (Fisman et al., 2022). More specifically, the unvaccinated people are expected to heighten the virus transmission in unvaccinated populations, however, due to the transmissible nature of COVID-19, the vaccinated populations also pose a risk of getting the infection, especially when herd immunity is only partially achieved. Besides, studies also reported that vaccinated people were much less likely to develop any range of common long-COVID symptoms, as compared to unvaccinated people when they are infected with the virus (Kuodi et al., 2022). Thus, governments must provide effective interventions to encourage voluntary vaccination as a collaborative action to halt the pandemic and achieve the best balance between values of public health and individual choice.

### COVID-19 around the world

The novel coronavirus outbreak was initially discovered in December 2019 in the Chinese city of Wuhan. On 11 February 2020, the new virus was named severe acute respiratory syndrome coronavirus 2 (SARS-CoV-2) by the International Committee on Taxonomy of Viruses (ICTV). Following the guidelines provided by the World Organisation for Animal Health (OIE) and the Food and Agriculture Organization of the United Nations (FAO), the World Health Organization (WHO) officially termed the virus ‘COVID-19’ (World Health Organisation, 2020). Its global spread began to dominate world news and, on 12 March 2020, the WHO declared there to be a global pandemic (World Health Organisation, 2020).

As a type of virus, there are many coronaviruses, some of which cause disease, some do not. First identified in 2019, SARS-CoV-2 (more commonly referred to as COVID-19) has caused a respiratory illness pandemic (John Hopkins Medicine Organization, 2021). COVID-19 can be spread through droplets, as well as from virus particles released into the air when an infected person breathes, talks, laughs, coughs, sings, or sneezes. While larger particles may fall to the ground in a few seconds due to their weight, tiny particles can instead linger in the air and accumulate within indoor spaces, particularly in crowded and poorly ventilated places (John Hopkins Medicine Organization, 2021). Common symptoms include a dry cough, fever or chills, shortness of breath or difficulty breathing, muscle or body aches, sore throat, loss of taste or smell, diarrhea, headache, fatigue, nausea or vomiting, and congestion or a runny nose. Some people may experience only mild symptoms, some could be asymptomatic, while others could require hospitalization (John Hopkins Medicine Organization, 2021).

As of 3 March 2022, there have been 438,968,263 confirmed COVID-19 cases worldwide, with a total of 5,969,439 deaths reported (World Health Organization, 2021). The pandemic has caused devastating effects in both developed and developing countries across the world.

### COVID-19 situation in Malaysia

Malaysia reported its first incidence of COVID-19 on 25 January 2020, which was linked to three Chinese citizens who had previously had close contact with an infected individual in Singapore (BERNAMA, 2020; New Straits Times, 2020). They entered Malaysia *via* Johor from Singapore on 23 January 2020. The three Chinese citizens were treated in Sungai Buloh Hospital in Selangor, Malaysia (New Straits Times, 2020). Meanwhile, a 41-year-old man was reported as the first Malaysian to test positive for COVID-19, following his return from Singapore. He started to develop a fever and cough and consequently sought medical treatment at a private health facility. Thereafter, he was referred to the Sungai Buloh Hospital, Selangor, and was admitted to an isolation ward (BERNAMA, 2020). The number of reported COVID-19 cases progressed at a slow rate in Malaysia until it reached its peak after a 4-day Tabligh Jamaat event conducted between 27 February 2020 and 1 March 2020 with over 16,000 participants at Kuala Lumpur’s Masjid Jamek Sri Petaling. This Tabligh’s cluster was declared over on 8 July 2020 after 3,375 individuals tested positive for COVID-19, resulting in 34 fatalities (Sheikh Yahya, 2020).

Cases of the virus continued to rise, posing risk to almost everyone in Malaysia. As a safety measure, the Malaysian government imposed a nationwide lockdown, known as the Movement Control Order (MCO), beginning on 18 March 2020. The adherence to the MCO showed the solidarity and civic-mindedness of Malaysian citizens, thus displaying their admirable qualities under the motto #KitaJagaKita (Sheikh Yahya, 2020). Eventually, Malaysia achieved a milestone when it reported zero COVID-19 local transmission cases on both 1 and 8 July 2020. It was, however, only short-lived, as the D614G mutations have been identified in European nations. This mutation was found to be 10-times more infectious than previous ones (Astro Awani, 2020). This new variant of concern, namely Omicron, looms large into 2022, which further ramp up the government’s calls for booster administration (Arumugam, 2022).

As COVID-19 continues to evolve, so does the development of its solution—which is to say its vaccines—that are designed to protect the community by reducing transmission. This type of protection is known as herd or community immunity (Radcliffe, 2021). Following the successful development of the COVID-19 vaccines, the world’s first dose was administrated to Margaret Keenan, a 91-year-old woman from the United Kingdom, on 8 December 2020 (PA Media, 2021). Meanwhile in Malaysia, the former Malaysian Prime Minister, Tan Sri Muhyiddin Yasin, became the country’s first person to receive the COVID-19 vaccine on 24 February 2021 (Parzi, 2021). According to the National COVID-19 Immunization Programme, the COVID-19 vaccination in Malaysia will be rolled out in three phases. The Special Committee for Ensuring Access to Covid-19 Vaccine Supply [JKJAV], 2021):

1) Phase 1 from February 2021 to April 2021, is dedicated to priority group 1 (e.g., Frontliners consisting of public and private healthcare personnel) and priority group 2 (e.g., Frontliners consisting of essential services, defense, and security personnel);
2) Phase 2 from April 2021 to August 2021, is dedicated to priority group 1 (e.g., remainder of healthcare workers, as well as those in essential services, defense, and security personnel) and priority group 2 (e.g., senior citizens aged 60 and above, high-risk groups with such chronic diseases as heart disease, obesity, diabetes, and high blood pressure, and people with disabilities);
3) Phase 3 from May 2021 to February 2022, is dedicated to the adult population aged 18 and above (both citizens and foreign nationals) with priority given to those in red zone areas, followed by yellow zones, and finally green zones.

It is reasonable to expect that only unprecedented collective action by states on a national and global scale can end the COVID-19 pandemic. Besides preventive protection measures, the long-term success of the public health response to the COVID-19 pandemic will depend on the population’s immunity. Accordingly, vaccination is among the key strategies with which to limit the spread of the virus, and improve health outcomes and life expectancy (Harrison and Wu, 2020). As the virus continued to grow progressively, so too did the development of vaccines. Indeed, such was their rapid progress that, to date, almost every country has either begun or completed its vaccination programs. The Malaysian government outline its target of vaccinating 80 percent of the country’s total population, approximately 33 million people, to achieve herd immunity.

As of 3 March 2022, a total of 25,755,241 (78.9%) of the total Malaysian population has been fully vaccinated, but only 14,884,826 (45.6%) people have received their booster shot. Health officials predicted that Malaysia will achieve herd immunity on 30 September 2022 with the vaccination of 26,125,920 individuals (Ministry of Health Malaysia, 2021). Remarkably, Malaysia has successfully achieved herd immunity among its adult population (those aged 18 and above) on 18 September 2021 with 18,738,358 vaccinated individuals and has been projected to reach 100 percent vaccination of its adult population on 31 December 2022 (Ministry of Health Malaysia, 2021). While there is evidence to show that herd immunity has already been reached among the adult population, those still unvaccinated should certainly not be left behind. The question as to whether these unvaccinated individuals should be categorized as conspiracy theorists, hesitant, or medically unfit to receive the vaccines remains a topic for debate.

Many factors have been shown to affect vaccine acceptance, including trust in the government (Lazarus et al., 2020). This has led to another main challenge: susceptibility to misinformation. Much virus information has been disseminated around various media (Wirawan et al., 2021), especially through social media platforms, which has either been false or only contains partial truth. Consequently, a considerable amount of the population began endorsing conspiracy theories. While the majority of the world’s population patiently awaits the arrival of the COVID-19 vaccine and is thrilled to accept it, there is a vocal minority that seeks to ‘explain’ the pandemic with conspiracy theories. Unfortunately, Muslim people are not exempt from these temptations. Belief in conspiracy theories is by no means a new phenomenon, and prior studies have highlighted some factors that might lead people to become more susceptible to conspiracies, such as feeling disconnected from society, unhappy or dissatisfied with one’s current life situation, those who have only a subjective worldview, who do not feel in control of their life, and those highly influenced by social media (Goreis and Voracek, 2019). While the probability that one may endorse conspiracy theories are minimal, but not impossible. Each individual—Muslims included—might have different reasons to hold a conspiracy belief. Among these reasons is a mistrust of science or governmental structures (Imhoff and Bruder, 2014; Richey, 2017), or a growing suspicion (or paranoia) that people are trying to control them (Richey, 2017).

Furthermore, as a response to curb the COVID-19 transmission, the enforcement of the MCO in Malaysia has witnessed a significant change in the community. Aspects of Islamic tradition and Muslim culture, such as visiting the sick and caring for the elderly, have either faded away or been placed on hold due to the lockdown measures (MediaCorp News Group, 2021). While a majority of people have struggled with social distancing and trying to adapt to the new situation, the rapid spread of the virus has demanded more strict measures, meaning that the government decided to close almost every mosque (Hassan, 2020; Yaacob, 2021). While these situations were deemed necessary, some people may take the view that they have been robbed and are trying to be controlled by others (Mokhtar, 2021). Consequently, they lose trust in the government and are highly likely to turn to conspiracy theories—which have in turn been proven to be negatively associated with vaccine acceptance (Jolley and Douglas, 2014; Earnshaw et al., 2020; El-Elimat et al., 2021).

The COVID-19 pandemic shares the same characteristic as other pandemics, namely an association with feelings of fear, anxiety, and worry (El-Elimat et al., 2021). Since the outbreak, most people are not only worried about infection, but also societal and economic concerns due to the preventative measures enforced by the government. These measures include social distancing and isolation, the closure of state and national borders, travel restrictions, the closure of education institutions (e.g., schools and universities), as well as the enforcement of curfews and lockdowns. Undeniably, further from the suffering caused by infection, the pandemic has also impacted people’s psychological and mental well-being. In Malaysia, the pandemic has been reported to have contributed to increased levels of anxiety, depression, and insomnia among the population (Mustapha, 2020). Moreover, it is also been found that anxiety, fear, and individual risk are important determinants of vaccine acceptance (Bendau et al., 2020). Contrary to conspiracy beliefs, individuals with higher levels of anxiety and risk perception show significantly higher vaccine acceptance (El-Elimat et al., 2021; Hao et al., 2021; Szmyd et al., 2021).

Besides, as cases and death toll escalate, the pandemic has also caused major disruption and shift in our daily life, especially with the extreme measures to contain the spread of the diseases across regions. Thus, it is not surprising to see a growing number of people who turned to endorse conspiracy beliefs. However, life may not always turn out to be as planned. Like a road, it has many bends, ups and down, from everyday struggles to traumatic events with massive impacts, such as life-altering events, the death of a loved one or a serious illness. People respond differently to each change, yet most of them generally adapt well over time to such stressful events–partly due to resilience. Resilience is widely referred to as “adversity or risk paired with positive outcomes” (Vella and Pai, 2019). In other words, it is “the process of adapting well in the face of adversity, trauma, tragedy, threats or significant sources of stress-including family and relationship problems, health problems, or workplace and financial stressors” (American Psychological Association, 2012). The presence of COVID-19 has impacted and challenged almost every people in many ways. Since resilience involves “bouncing back” from challenging experiences or struggles, it may also lead to significant personal growth. While people’s attitude toward vaccination may be contributed by various factors–such as anxiety and conspiracy beliefs, individual’s response to vaccination may be different according to their level of resilience.

Although confidence in vaccines continues to rise, vaccine hesitancy and unwillingness remain a major concern to many countries—as are conspiracy theories. Therefore, it is crucial to understand the underlying factors behind vaccine acceptance. Since most previous studies have been conducted in Western countries, there is an urgent need for a nuanced understanding of attitudes toward COVID-19 vaccines in the East—in this case, among the Malaysian population with a focus on Muslim individuals. In so doing, the most effective strategies can be identified to increase vaccine coverage to bring the pandemic to an end. Therefore, the current study seeks to investigate the effect between anxiety, conspiracy theories and individuals’ resilience on Muslim people’s attitudes toward the COVID-19 vaccines. Moreover, owing to the scarcity of literature comparing male and female behaviors, we also seek to explore whether disparities between gender exist in explaining vaccination attitudes.

In this paper, we addressed five objectives:

• To examine the relationship between COVID-19-related anxiety and their attitude toward vaccines among Malaysian Muslims.
• To examine the relationship between conspiracy beliefs and their attitude on vaccines among Malaysian Muslims.
• To examine the relationship between individuals’ resilience and their attitude toward vaccines among Malaysian Muslims.
• To investigate the moderating role of an individual’s resilience in explaining the relationship between conspiracy beliefs and attitudes toward vaccines.
• To investigate disparities in interrelationships between variables under study among female and male groups.

## Theoretical background and hypothesis development

To address the research gap, the study adopted the stimulus-organism-response (S-O-R) framework, owning to its predictive power to evaluate consumer response to environmental stimuli which was evident in previous studies (Mehrabian and Russell, 1974). The S-O-R model is made up of three components; stimulus, organism, and response, which decide the behavioral outcome of an event (Skinner, 1935). The stimulus is an environmental trigger that arouses consumers and affects the psychological state of an individual (Young, 2016). It can be both internal and external. In essence, internally, consumers are motivated by their own emotions, perceptions, and mental formations, which is a psychological process that also generates the signal for selection, sense-making and interpretation. External stimuli, on the other hand, may be both tangible and intangible (Chai et al., 2019).

Meanwhile, the organism represents the consumers’ internal evaluations, which consist of the affective and cognitive states. Affective states refer to emotional responses when an individual interacts with the environment, whereas cognitive states refer to mental processes that occur when an individual interacts with the stimuli. The organism reflects the consumers’ perceptions, judgment, emotions, motivations and thinking, resulting from an experiential encounter with the stimulus (Chai et al., 2019). In addition, the organism also involves consumers’ positive and negative experiences, as a result of their purchase response. In this sense, the S-O-R model is not only linear, but it also allows for feedback-loop influence, and in some cases, allows “S” to directly affect “R” while bypassing the “O” (Chai et al., 2019). Finally, the third element of the S-O-R model is the response, which refers to an individual’s behavioral outcome that may be positive or negative (Donovan and Rossiter, 1982).

In the context of COVID-19, the study suggests that the stimulus should reflect the conspiracy beliefs from which individuals engage during the pandemic. The organism in this study represents anxiety; which is a natural adaptive emotion that promotes survival by prompting a person to avoid or flee a potentially dangerous situation (Crocq, 2015), and individuals’ resilience; which is an ability to cope effectively in response to stressful or traumatic events involving risk or adversity (Masten, 2001; Bonanno, 2012), while, the attitude toward COVID-19 vaccine represents as a response.

Furthermore, we used conceptual reasoning from Protection Motivation Theory (PMT) (Rogers, 1975) to establish causality between relevant constructs. The theory describes individuals’ motivation to engage in protective behaviors in the presence of a threat stimulus (Rogers, 1975). The decision to engage in protective behaviors are governed by two cognitive processes; threat appraisal and coping appraisal (Rogers, 1983). Individuals use threat appraisal to assess the level of threat, while a coping appraisal is the capability of an individual to carry out protective behaviors in the presence of a threat (Rogers, 1983). In the present study, the PMT is used to better understand potential predictors of individuals’ attitudes toward COVID-19 vaccinations. Hence, we hypothesized:

H1. COVID-19-related anxiety has a positive influence on individuals’ attitudes toward vaccination.

H2. Conspiracy belief has a negative influence on individuals’ attitudes toward vaccination.

H3. Individual resilience has a positive influence on individuals’ attitudes toward vaccination.

H4. The relationship between conspiracy beliefs and vaccination attitude is weakened for individuals with a higher level of resilience.

H5. The strength of the relationship between COVID-19-related anxiety and individuals’ attitude toward vaccination is different between female and male.

H6. The strength of the relationship between conspiracy beliefs and individuals’ attitudes toward vaccination is different between female and male.

H7. The strength of the relationship between individual resilience and individuals’ attitude toward vaccination is different between female and male.

H8. The strength of the relationship between conspiracy beliefs and individuals’ attitudes toward vaccination which is moderated by individual resilience is different between female and male.

**FIGURE 1:**
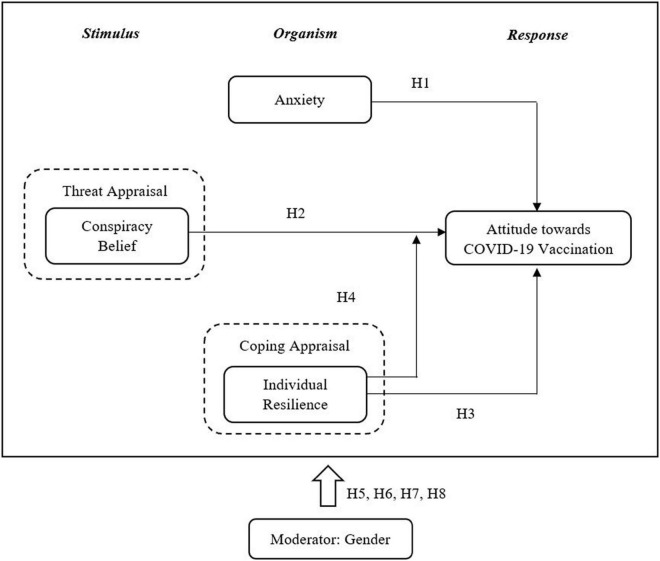
Conceptual framework.

In general, the correlations indicated above can be seen in Figure 1, the conceptual framework.

## Methods

### Study design and data collection procedure

This cross-sectional study employed convenience sampling to collect the data. Convenience sampling, also known as haphazard or accidental sampling, is a non-probability sampling method which involves selecting a target population based on practical criteria, such as easy access, geographical proximity, availability at a given time, or the readiness to participate (Etikan, 2016). We chose this sampling method as it does not require any underlying theories or a fixed number of participants.

For this study, our target respondents for the research questionnaire survey were all members of the Malaysian Muslim public. The target sample’s main criterion was: (1) aged 18 and above; (2) a follower of the Muslim faith; and (3) currently residing in Malaysia. Muslim samples were chosen due to several reasons. First, Malaysian Muslims were chosen as they were the largest religious group and most broadly practiced religion in Malaysia with approximately 63.5 percent (20.6 million people) (Department of Statistics Malaysia, 2022), which implies that Muslims are the majority community in Malaysia. Thus, it will be worth understanding the behaviors and responses of Muslim segments to COVID-19, especially concerning vaccination as they make up a huge segment in Malaysia. In order to ensure the government formulate the best strategy for encouraging the COVID-19 vaccination program, it is certainly essential to understand the perspective of the major population.

Secondly, due to the pandemic, Muslim communities have faced a variety of religious challenges, such as suspensions of their primary religious duties, including pilgrimage, the Friday prayers, and congregations, to save people from the pandemic’s deadly consequences (Abbasi et al., 2021). Moreover, the pandemic also restricted Muslims from practicing their traditions such as visiting elderly families, eating together, sharing utensils, or intimate social behaviors (e.g., kissing the hand or head of elderly family members) (Ahmed et al., 2020; Ozalp, 2020). Because of these practices, some Muslims may find present COVID-19 interventions and protective behaviors as ‘alien and absurd’ (Ahmed et al., 2020; Hussain, 2020; Ozalp, 2020). Furthermore, according to health professionals, Muslims are among the most vulnerable populations or segments due to a variety of cultural factors (Hussain, 2020). It is reported that in some countries, Muslims are to be blamed for the increased prevalence of COVID-19 cases throughout the community (Jha and Dixit, 2020; Rahim, 2020), and a similar situation was also faced by Muslims in Malaysia (Adetunji, 2020; Ananthalakshmi and Sipalan, 2020). During the pandemic, collective voluntary responses are needed, and this blaming situation may be harmful to subgroups’ willingness to participate in governments’ initiatives (Bouguettaya et al., 2022). Besides, it may also lead to varying consequences, both directly on the COVID-19 response and indirectly by promoting unfavorable and intolerable general social issues (Bouguettaya et al., 2022). Thus, the decision to choose Muslims as the study’s respondent was considered an acceptable option.

We distributed a self-administered questionnaire to the target respondents *via* a survey questionnaire. Since this study has been conducted during the COVID-19 pandemic, the questionnaires were developed and distributed online by using Google Forms for data collection, reducing contact and face-to-face communication. During the COVID-19 pandemic, it was not feasible to distribute the questionnaire face-to-face due to the risk of secondary infections (Hur and Chang, 2020). Thus, we deemed an online distribution more appropriate as it also allows the more rapid collection of behavioral data when fieldwork is unavailable (Hur and Chang, 2020).

In order to improve the number of responses, we adopted several measures, including promoting and sharing the survey through community pages and such social media platforms as Facebook and TikTok, as well as communication platforms, such as WhatsApp and Telegram. We deemed these measures appropriate as the abovementioned social media platforms were reported as being among the most preferred social networking and communication applications in Malaysia (Malaysian Communications and Multimedia Commission, 2020). Moreover, during Malaysia’s MCO, Telegram became a medium for learning and a popular platform on which to disseminate official government information, which further demonstrated its suitability for our data collection purposes.

According to Hair et al. (2006), a sample size ranging between at least 150 and 400 respondents should be sufficient for structural equation modeling. While, Reinartz et al. (2009) on the other hand, have identified 100 samples as the sampling threshold for PLS-SEM. Nonetheless, this study employed *a priori* estimation to estimate sample size by using power analysis. The minimum sample size is determined by power analysis, which recognizes the part of a model with the largest number of predictors (Hair et al., 2014a; Memon et al., 2020). We used the G*Power calculation for two mean formulas to estimate the minimum sample required (Faul et al., 2007; Erdfelder et al., 2009). The estimated minimum samples of 92 cases were recommended in order to achieve a statistical power of 0.8. A total of 438 completed and valid questionnaires were collected and used for further analyses. Therefore, as our sample size (438) was greater than that recommended minimum samples, it can be considered acceptable.

The respondents’ demographics are presented in Table 1.

**TABLE 1:** Sample characteristics.

Variable	Categories	Frequency	Percentage
Gender	Male	205	46.8
	Female	233	53.2
Age	<24	88	20.1
	25–40	119	27.2
	41–56	117	26.7
	57–66	58	13.2
	67–75	56	12.8
Marital Status	Single	97	22.1
	Married	341	77.9
Education	Master’s Degree	4	0.9
	Bachelor’s Degree	98	22.4
	Diploma/Executive	97	22.1
	Certificate		
	STPM	94	21.5
	SPM	99	22.6
	PMR	46	10.5
Employment Status	Student	86	19.6
	Unemployed	3	0.7
	Self-employed	72	16.4
	Home maker	2	0.5
	Public sector	60	13.7
	Private sector	124	28.3
	Retired	91	20.8
Monthly Income	<RM2000	32	7.3
	RM2000–3999	69	15.8
	RM4000–5999	157	35.8
	RM6000–7999	130	29.7
	RM8000–9999	24	5.5
	>RM10000	26	5.9

### Study instruments

The questionnaire consisted of four main sections. The first addressed the respondents’ demographic information. The second contained questions pertaining to COVID-19-related anxiety, followed by, third, belief in conspiracy theories, fourth, individual’s resilience and, fifth, attitudes toward COVID-19 vaccines. The survey was offered in both English and Malay languages. In order to ensure and maintain linguistic and conceptual equivalence, we employed a backward-translation approach (Brislin, 1970). We rectified any discrepancies found during the process and validated them by consulting with bilingual researchers.

All the instrument constructs were adopted from the established sources in the area of study. We measured COVID-19-related anxiety by adopting Lee’s Coronavirus Anxiety Scale (2020). In past studies, these instruments have been tested among various ethnicity; namely White, Blacks, Hispanics, and Asians, and it has been reported to measure anxiety symptoms in similar ways across demographic groups (Lee, 2020). Each item was written to encapsulate a distinct manifestation of COVID-19 related anxiety. We used a five-point scale anchored from (0) ‘not at all’ to (4) ‘nearly every day’ to reflect the frequency of the symptoms that the respondents are experiencing over preceding 2 weeks.

Meanwhile, we adopted instrument items regarding conspiracy beliefs from Freeman et al. (2020). This instrument has been tested among various public groups including representatives from ethnic minority groups (e.g., White and Black Caribbean, White and Black African, White and Asian, Indian, Pakistani, Bangladeshi, Chinese, and Arab) (Freeman et al., 2021). We measured the responses with a five-point scale ranging from (1) ‘strongly disagree’ to (5) ‘strongly agree.’ Higher scores indicate greater endorsement of coronavirus conspiracy beliefs.

To investigate vaccine attitudes, we adopted the SAGE Vaccine Hesitancy Survey (2014). These includes whether respondents feel that taking COVID-19 vaccine as a good idea, whether they have a positive view toward receiving COVID-19 vaccine, whether they will encourage their family, friends and relatives to get vaccinated, and whether they perceived taking COVID-19 vaccine as important. In the previous study by Kumar et al. (2021), these instruments has been tested and validated among people which came from various ethnicity, including, Qatari, Arab-non-Qatari, Asian, African, European, North America, Central America, and South America. Similarly, we used a five-point scale ranging from (1) ‘strongly disagree’ to (5) ‘strongly agree’ to measure the responses.

To assess the individual’s resilience, we adopted the brief resilience scale (BRS) from Smith et al. (2008), which measured the individual’s perceived ability to bounce back or recover from stress. The instrument has been validated in past studies, it has been tested and used in various populations from countries such as Germany (Kunzler et al., 2018), Spanish (Rodríguez-Rey et al., 2016), Netherland (Soer et al., 2019), Mexican and Chilean (Hidalgo-Rasmussen and Gonzalez-Betanzos, 2019). Likewise, we also used a five-point scale ranging from (1) ‘strongly disagree’ to (5) ‘strongly agree’ to measure the responses. For the individual resilience construct, the average score for each response is interpreted as follows; low scores (between 1.00 and 2.99) indicate low psychological resilience, medium scores (between 3.00 and 4.30) indicate normal resilience, while high scores (between 4.31 and 5.00) indicate high psychological resilience.

### Data analysis

In order to assess the research model, we used the variance-based partial least square structural equation modelling (PLS-SEM). We selected PLS-SEM for a number of reasons. First, it has the ability to simultaneously analyze every association between the constructs in a conceptual model, as well as being able to support a multi-group analysis (Henseler et al., 2016; Hair et al., 2017). Furthermore, the analysis demonstrated that the data were not multivariate normal—Mardia’s multivariate skewness (β = 4.52, *p* < 0.01) and Mardia’s multivariate kurtosis (β = 18.35, *p* < 0.01). Hence, the application of a non-parametric analysis, such as PLS-SEM, was highly appropriate for a study such as this (Chin et al., 2013). We employed SmartPLS 3.0 software to analyze the data, and we reported the findings according to guidelines provided by Hair et al. (2017) and Ramayah et al. (2018).

## Results

### Descriptive analysis

A total of 438 respondents completed the survey, of which 312 (71.2%) had not received their COVID-19 vaccination, while another 126 (28.8%) had, but they yet to complete their second dose. It has been reported that, in some instances, individuals purposefully missed their scheduled second dose due to several reasons, such as fears of the side effects and their perception that they were sufficiently protected with a single shot (Robbins, 2021). A similar situation has been found in Malaysia, where several states (e.g., Melaka, Perak, Labuan, and Kelantan) reported thousands of individuals failing to attend to their second vaccination appointments (BERNAMA, 2021; Mohd Nawi, 2021; Zahari and Mamat, 2021). Thus, taking this into consideration, we opted to further analyze both categories so as to investigate their attitudes toward COVID-19 vaccinations.

The respondent’s demographic profile is presented in Table 1. There were 205 (46.8%) male and 233 (53.2%) female respondents, where the majority were aged between 25–40 (27.2%), 41–56 (26.7%), and below 24 (20.1%). The majority of the respondents were married (77.9%) and approximately 66.9% had completed their tertiary education (i.e., STPM, Diploma/Executive Certificate, bachelor’s degree, and master’s degree). The respondents worked in various sectors and capacities, such as the private sector (28.3%), self-employed (16.4%), and the public sector (13.7%). Most of the respondents also earned between RM4000–5999 (35.8%), and RM6000 to RM7999 (29.7%) per month.

### Common method variance

Since the study adopted single-source data, we had to ensure that the results were free from bias. To do so, we needed to address the common method variance (CMV) prior to the analysis (Mackenzie et al., 2011). To confirm that CMV was not a severe issue in the study, we conducted a full collinearity analysis (Kock, 2015). Generally speaking, if the variance inflated factor (VIF) is greater than or equal to 3.3, the study suffers from CMV (Kock, 2015). Table 2 demonstrates that the VIF values were lower than 3.3, suggesting that the CMV was not a severe issue in the present study.

**TABLE 2:** Full collinearity.

Construct	VIF
Anxiety	1.007
Conspiracy Beliefs	1.007
Resilience	1.001

### Measurement model evaluation

The study employed a two-staged approach for conducting the analytical procedure. We first evaluated the measurement model to assess the instruments’ reliability and validity. Second, we further evaluated the structural model to assess the hypothesized association between the constructs (Anderson and Gerbing, 1988).

We assessed the convergent validity and discriminant validity for the reflective constructs. According to Hair et al. (2017), convergent validity is confirmed if several conditions are met: the loading of all indicators is greater than or equal to the 0.708 thresholds, the average variance extracted (AVE) is greater than or equal to the 0.50 threshold, and the composite reliability (CR) is greater than or equal to the 0.70 thresholds. Only items that met the required criteria were retained. Table 3 presents the results of the convergent validity for all items. As shown, all values for loading, AVE, and CR were greater than their threshold values, indicating the convergent validity of all latent variables.

**TABLE 3:** Reliability and convergent validity.

Construct	Item	Loading	CR	AVE
Attitude	Attitude1	0.916	0.916	0.733
	Attitude2	0.762		
	Attitude3	0.868		
	Attitude4	0.871		
Anxiety	CAS1	0.730	0.708	0.548
	CAS2	0.750		

Additionally, researchers are required to use the heterotrait-monotrait (HTMT) ratio of correlations in order to establish discriminant validity (Franke and Sarstedt, 2018). Discriminant validity is confirmed when all HTMT values are smaller than or equal to 0.85. Table 4 shows that all of the HTMT values were lower than 0.85, meaning that the discriminant validity was established.

**TABLE 4:** Discriminant validity—HTMT.

	Anxiety	Attitude
**Anxiety**		
Attitude	0.261	

Meanwhile, for the formative constructs, we assessed the convergent validity through the evaluation of the collinearity—variance inflation factor (VIF), the significance of weight and loading for each item (Hair et al., 2017). Only items that met the criteria were retained. Table 5 shows that all VIFs were lower than the threshold value of 3.3 (Diamantopoulos and Siguaw, 2006), and all the outer weights and loadings were significant, thus confirming the convergent validity of the formative measurement model.

**TABLE 5:** Convergent validity (formative construct).

Construct	Item	Weight	VIF	*T*-Value Weight	*T*-Value Loading
Conspiracy Beliefs	CCS1	0.535	1.029	3.566	2.583
	CCS2	0.288	1.027	1.839	1.990
	CCS3	–0.291	1.017	1.902	1.169
	CCS6	0.575	1.022	4.353	4.850
	CCS7	0.523	1.010	3.480	3.314
Individual’s Resilience	BRC2	0.720	1.000	2.868	2.811
	BRC4	0.467	1.003	1.624	1.712
	BRC6	0.499	1.003	1.735	1.819

### Structural model evaluation

To assess the study’s hypotheses, we applied a bootstrapping procedure with 5,000 samples. The hypotheses were supported if the beta value was in line with the direction of the hypothesis, the *t*-value was greater than or equal to 1.645, the *p*-value was less than or equal to 0.05, and there was no zero straddle between the lower level (LL) and upper level (UL) of the confidence interval. Table 6 presents the results of the hypotheses tested in the study. As can be seen, all of the hypotheses were supported.

**TABLE 6:** Hypothesis testing.

Hypothesis	Relationship	Beta	SE	*T*-Value	*p*-Values	LL	UL	Decision	*R* ^2^	*Q* ^2^	*f* ^2^	VIF
H1	Anxiety → Attitude	0.077	0.043	1.791	0.037*	0.015	0.150	Supported	0.151	0.090	0.007	1.007
H2	Conspiracy Beliefs → Attitude	–0.276	0.044	6.248	0.000***	–0.347	–0.202	Supported			0.089	1.007
H3	Individual’s Resilience → Attitude	0.138	0.045	3.073	0.001**	0.066	0.210	Supported			0.022	1.001
H4	Conspiracy Beliefs x Attitude → Individual’s Resilience	–0.205	0.123	1.671	0.047*	–0.331	0.183	Supported			0.051	1.001

***p < 0.001; **p < 0.01; * p < 0.05.

The results revealed that anxiety has a positive effect on COVID-19 vaccination attitudes, thus supporting H1 (β = 0.077, *p* = 0.037). Likewise, the results showed that belief in conspiracy theories has an inverse effect on vaccination attitudes, thereby supporting H2 (β = −0.276, *p* = 0.000). Similarly, the results showed that an individual’s resilience has a positive effect on vaccination attitudes, thus supporting H3 (β = 0.138, *p* = 0.001). Furthermore, it is also found that the relationship between conspiracy beliefs and vaccination attitude is weakened for individuals with a higher level of resilience (β = −0.205, *p* = 0.047), thereby supporting for H4.

We subsequently assessed the coefficient of determination (R^2^), the effect size (f^2^), and the predictive relevance (Q^2^). Coefficient of determination represents a model’s predictive accuracy (Cohen, 1988). A higher R^2^ value indicates greater explanatory power, which ranges from 0.75 (large), 0.50 (moderate), and 0.25 (small). We found an *R*^2^ value of 0.151 for attitude, suggesting that anxiety, conspiracy beliefs and an individual’s resilience constituted 15.1% of the variance in the attitude. On the other hand, effect size (f^2^) demonstrates the statistical significance of the measures. The f^2^ value ranges from 0.35 (large), 0.15 (moderate), and 0.02 (small) (Cohen, 1988), alternatively the interpretation of f^2^ may also follow Kenny’s (2016) guidelines, whereby 0.025 (large), 0.01 (moderate), and 0.005 (small). Table 6 shows that anxiety has a small effect size, whereas conspiracy belief has a large effect size and an individual’s resilience has a moderate effect size.

Last, but by no means least, the predictive relevance (Q^2^) is a measure to evaluate the predictive relevance of the inner model (Hair et al., 2014b). A value greater than zero indicates the predictive relevancy of the model, while a value of less than zero indicates a lack of predictive relevancy (Henseler et al., 2015). Table 6 shows that the Q^2^ value of the endogenous construct was less than zero, thereby depicting a lack of predictive relevancy of the model.

### Assessment of group differences

We conducted PLS multi-group analysis (MGA) to test the differences between the male and female datasets. Before doing so, however, we had to first confirm the measurement invariance of composite (MICOM) (Henseler et al., 2016; Hair et al., 2017). Table 7 presents the partial measurement invariance on all constructs for both male and female groups, suggesting the need to compare differences between male and female groups using MGA. Table 8 illustrates the results of the hypotheses testing for a dataset split by gender.

**TABLE 7:** Results of invariance measurement testing.

Constructs	Configural Invariance	Compositional Invariance (Correlation = 1)		Partial Measurement Invariance Established	Equal Mean Value		Equal Variance		Full Measurement Invariance Established
		C = 1	5% quartile of the empirical distribution of c		Differences	Confidence Interval	Differences	Confidence Interval	
Anxiety	Yes	0.973	0.680	Yes	–0.092	−0.157, 0.159	0.032	−0.184, 0.188	Yes
Attitude	Yes	0.999	0.999	Yes	0.131	−0.156, 0.155	0.423	−0.381, 0.393	No
Conspiracy Beliefs	Yes	0.799	0.787	Yes	0.058	−0.157, 0.155	–0.107	−0.203, 0.213	Yes
Individual’s Resilience	Yes	0.691	0.593	Yes	0.077	−0.163, 0.161	–0.007	−0.205, 0.205	Yes
Anxiety × Attitude → Individuals’ Resilience	Yes	0.301	0.044	Yes	0.089	−0.159, 0.159	0.157	−0.313, 0.326	Yes

**TABLE 8:** Results of hypotheses testing for multigroup analysis.

Hypothesis	Relationship	Path Coefficient		*P*-value			Decision	Result
		Female	Male	Permutation	MGA	Welch-Satterthwait Test		
H5	Anxiety → Attitude	0.057	0.124	0.192	0.218	0.223	Not supported	F = M
H6	Conspiracy Beliefs → Attitude	–0.250	–0.277	0.385	0.439	0.413	Not supported	F = M
H7	Individual’s Resilience → Attitude	0.224	–0.005	0.009*	0.012*	0.019*	Supported	F≠M
H8	Conspiracy Beliefs × Attitude → Individual’s Resilience	–0.257	0.360	0.011*	0.017*	0.076	Supported	F≠M

A p-value of MGA lower than 0.05 or higher than 0.95 indicates significant difference at the 5% level. Permutation and Welch-Satterthwait tests are significant for p-value only at the 5% level, which is lower than 0.05. *p < 0.05 or p > 0.95.

It is found that there is a significant difference between female and male groups for the relationship between individual’s resilience and attitudes toward COVID-19 vaccination (H7), and the relationship between conspiracy beliefs and attitudes toward vaccines that is weakened for individuals with a high level of resilience (H8). Thus, H7 and H8 were supported. However, contrary to our expectations, we found no significant difference between female and male groups for the relationship between anxiety and attitude toward COVID-19 vaccination (H5), and conspiracy beliefs and attitudes toward the vaccines (H6). Therefore, H5 and H6 were unsupported.

## Discussion and implications

### Theoretical implications

The present study sought to assess whether, in Malaysia, anxiety, the belief in conspiracy theories and individual resilience affect peoples’ attitudes toward COVID-19 vaccination, and whether these differed by gender. The analysis revealed that anxiety has a positive relationship with vaccination attitudes, thus corroborating the findings from previous studies which highlighted that individuals with higher risk perception and greater anxiety levels showed significantly higher vaccine acceptance in Turkey, the United Kingdom (Freeman et al., 2021), the United States (Bendau et al., 2020; Fisher et al., 2020), and France (Detoc et al., 2020). Interestingly, COVID-19-related anxiety instills a sense of fear within people and is consequently associated with a more positive or accepting attitude toward vaccination. This seems reasonable as fears associated with one’s physical health or that of loved ones are known to have the ability to foster stronger vaccine acceptance as a way of minimizing the likelihood of undesirable consequences. Furthermore, the growing number of daily confirmed cases and death rates caused by COVID-19, and especially the news coverage about many hospitals’ introduction of special containers with which to store the bodies of deceased patients (Bedi, 2021), has caused mixed emotions among the Malaysian population. However, it is important to note that the positive association between anxiety and vaccination attitudes should not necessarily be taken to mean that it is better to induce fears among people in order to increase vaccine acceptance. Rather, the present study instead aims to foreground the need to enhance risk communication and promote more effective preventive actions against the spread of COVID-19.

The analysis also revealed that belief in conspiracy theories was inversely associated with vaccination attitudes, which is consistent with prior studies that found people who endorsed conspiracy theories to be negatively related to vaccination uptake (Hornsey et al., 2018; Bertin et al., 2020; Earnshaw et al., 2020; Romer and Jamieson, 2020; Pivetti et al., 2021). This finding suggests that conspiracy theorists are less likely to be in favor of vaccines. Likewise, past studies suggest that the likelihood of vaccine intent will be reduced when an individual believed in conspiracy theories (e.g., COVID is man-made, business theory and population control theory) (Ghaddar et al., 2022). More often than not, the online health information provided is not always supported by scientific evidence as it is frequently bolstered with baseless rumors and conspiracy theories (Lavorgna et al., 2018). Besides, the implementation of any vaccine-related policy may be at risk when the distrust between government stakeholders and people in the community is heightened, especially when rumors regarding the delays of COVID-19 development, or the reachability of vaccines are specially reserved only for supporters of the ruling government (Akther and Nur, 2022).

Furthermore, the analysis also revealed that an individual’s resilience played an important role in the attitudes toward vaccination. The finding corroborates with previous studies which suggested resilience was associated with greater intent to receive COVID-19 vaccination (Mo et al., 2022). Since resilience is an important element in positive psychology, it has the ability to foster well-being and positive behaviors (Mo et al., 2022). As a positive emotionality, resilience encourages individuals to put behind the limitations of negative thoughts and undertake more novel and innovative ways of thought and action (Fredrickson and Joiner, 2002). Furthermore, promoting resilience is listed as one of the 10 most important considerations that needed attention in order to successfully manage COVID-19 globally (Habersaat et al., 2020). A high resilience individual is also more likely to experience a lower level of job burnout (Manzano García and Ayala Calvo, 2012), anxiety and depression rates (Manzano García and Ayala Calvo, 2012; Zhang et al., 2020). The reason why they can cope with psychological stress more effectively is contributed to their ability to maintain a positive attitude despite the life-threatening events (Guo et al., 2018).

It is also found that the relationship between conspiracy beliefs and vaccination attitudes was weakened for individuals with a high level of resilience. These findings corroborate with the current literature that suggests some individuals are more resilient, while some are more fragile, especially during traumatic events such as the COVID-19 pandemic (Panzeri et al., 2021; Valiente et al., 2021). A highly resilient individual is more likely to engage in different behavioral options (Fredrickson, 2001) and they are more likely to react positively to stressful events (Smith et al., 2017; Ay et al., 2021). Resilience will affect an individual’s capability to solve a problem and their ability to evaluate situations, as well as how they act in response to a situation (Polk, 1997). Individuals with higher resilience perceived COVID-19 as a lesser threat (Razzak and Yousaf, 2022) and they are less likely to believe in conspiracy threats (Miller, 2020). Consequently, a favorable attitude on COVID-19 vaccination can be viewed as a novel and positive coping strategy (Mo et al., 2022).

Additionally, the findings suggest differences between the effects of individual resilience, and attitudes toward vaccines across groups. The results indicate two relationships, firstly, the effect between an individual’s resilience and vaccination attitudes is higher for female groups. Interestingly, this is contrary to past studies that suggest that females had significantly lower resilience than males (Dai et al., 2020; Afshari et al., 2021). Literature has shown that women are more likely than men to perceive and focus on negativity, especially during a stressful event (Riecher-Rössler, 2017). This phenomenon has been demonstrated in several studies during the COVID-19 pandemic where women are shown to be psychological impacted more seriously than men (Özdin and Bayrak Özdin, 2020). Furthermore, women are also reported to experience symptoms of stress and anxiety more severely than men (Hou et al., 2020). The reason could be attributed to the biological difference between males and females, particularly in terms of how they view things and ways of approaches to solve certain issues during critical situations. However, there are also past studies which corroborate with our findings. Zarulli et al. (2018) in their study found that women are more resilient during a challenging period, and they are also thriving in greater numbers than men, although the overall mortality rates and lifespan were at lower rates. Besides, even though women are more likely to have poorer health status, which signifies that they are frailer, Gordon and Hubbard (2020) found that they have a longer life expectancy which signifies that they are more resilient. A better understanding of the role of gender is indeed important to ensure programs and initiatives related to public health are strategized effectively. Thus, mixed evidence on gender differences in response to the COVID-19 pandemic certainly calls for more empirical work.

Secondly, the inversed effect between conspiracy beliefs and vaccination attitudes are weakened for an individual with a higher level of resilience is also significantly different between female and male groups. The findings suggest that the male group scores higher in this relationship than the female group. Since men have a significantly higher tendency to endorse COVID-19 conspiracy belief (Cassese et al., 2020), thus the inversed effect between conspiracy beliefs and their attitudes toward vaccination are weakened and more apparent for men when their resilience are higher. These findings are in tandem with the prior research (Sambu and Mhongo, 2019), which suggests gender has a greater influence on an individual’s resilience. This may be contributed by the notion that both men and women have different personality trait–while women are more likely to communicate with others and consequently attained the required support to cope with the crisis, men on the other hand tend to communicate less especially during the time of crisis and they end up getting less help and empathy as compared to women (Sambu and Mhongo, 2019).

However, contrary to our expectation, we found that the relationship between anxiety and conspiracy beliefs with COVID-19 vaccination attitudes was not significantly different for both male and female groups. This result is consistent with past studies (Salali and Uysal, 2021). It should be noted that, although Malaysia still has traditional gender roles, where men are expected to be breadwinners and women caregivers (Lembaga Penduduk dan Pembangunan Keluarga Negara [LPPKN], 2019), the concept of shared responsibility is much more prevalent and acceptable among younger and older millennials (Lembaga Penduduk dan Pembangunan Keluarga Negara [LPPKN], 2019). This means that men are more likely to be involved in housework, childcare, and eldercare, whereas women are more likely to be engaged in active employment.

Millennials constituted 47.3% of our total respondents, making them the most prevalent generational group. Therefore, both male and female individuals may share similar or equal views regarding COVID-19 vaccination as both groups must work either outside or at home, but interact with people regardless. This aligns with the recent government calls to make COVID-19 vaccination mandatory among federal government employees (Latiff, 2021). Besides, for other employment sectors, while there is no specific mandatory vaccination policy, employers would generally be able to make it compulsory for their employees to be vaccinated so as to ensure the safety and health of all staff, as well as to curb the spread of the virus (Lim, 2021). In both cases, those who refuse vaccination could face disciplinary action due to their potential to cause loss or damage to the company or other employees, particularly when their job function requires them to have regular and frequent physical interactions with third parties (Lim, 2021).

### Practical implications

Two years after the outbreak of COVID-19, the increasing trend is one of acceptance of the vaccine, with a rate of 85.3 percent in 2021 as compared to 67 percent in 2020. Besides, refusal to take the vaccine is also a decreasing trend, with only 5 percent of people refusing in 2021 as compared to 16 percent in 2020. In 2021, a total of 95 percent of people agreed to take the vaccine because of government recommendations and efforts, compared to 83.3 percent in 2020 (Institute for Health Behavioural Research, 2021). The lower percentage in the preceding year may be attributed to the fewer availability of vaccines at the time. The majority of our respondents reported feeling confident that the vaccine could prevent infection, which corroborates findings from a survey conducted by the Institute of Health Behavioural Research (IHBR), Malaysia, whereby a total of 98.5 percent of people felt confident with vaccine effectiveness in 2021, as compared to 79.5 percent in 2020 (Institute for Health Behavioural Research, 2021).

Although there is a relatively small amount of people who are yet to be vaccinated, it would be complacent to assume that these small numbers will not do any harm. People are always at risk of becoming super-spreaders—which refers to those with a greater than average propensity to infect a larger number of people (Cave, 2020). This was evidenced by Case 26, Malaysia’s first super-spreader (Dorall, 2020), also famously known as the Tabligh cluster which infected a total of 1,143 other people (Lin, 2020), and the Sivagangga cluster from Kedah which resulted in approximately 200 positive cases (Danial et al., 2020; Tan, 2020), as well as the infamous Pasai cluster which infected a total of 2,693 other people (Chua, 2021; Dayak Daily, 2021).

According to the literature, among the reasons given by those who refused vaccination (particularly in Malaysia) are the fear of injections and concerns regarding the adverse effects of vaccines (Ahmad, 2021; Institute for Health Behavioural Research, 2021). Meanwhile, some firmly believe in the strength of their immune systems due to their lack of previous illnesses. Moreover, some elderly citizens have simply refused vaccination out of preference and a lack of necessity due to their being at the later stages of their lives (Ahmad, 2021). Surprisingly, some have even claimed that, if they refuse vaccination, they would not die since they have no plans to travel (Ahmad, 2021), and there are others who used religion to rationalize their refusal to receive the vaccine as they doubt its halal status (Institute for Health Behavioural Research, 2021; Syed Bakri, 2021). Additionally, some prefer to take the ‘wait and see’ approach, while others believe that vaccines are related to conspiracy theories and claim a hidden agenda (Syed Bakri, 2021). Furthermore, based on a survey conducted by the IHBR, National Institute of Health, Malaysia, 62.4 percent of the respondents expressed doubts over the vaccine’s brand and developer, 83.9 percent doubted whether the vaccine could offer protection from infection, and 80.7 percent claimed that the vaccine is not safe (Institute for Health Behavioural Research, 2021).

Undeniably, actions from various groups are required to ensure the success of the immunization plan. A vaccine alone cannot be the sole solution, but it could allow us to obtain the herd immunity necessary to drastically reduce infection and subsequent death. Consequently, the country will develop the capacity to control transmission and thus can guarantee that it will establish the most effective national health system. Numerous initiatives have been taken to ensure the success of the country’s immunization plan. One important initiative that requires intensified attention is the dissemination of continuous knowledge to nurture understanding among the community and counter the dangerous spread of misinformation. As the main decision maker, the government play an important role in strengthening vaccine literacy among the people. Nowadays, although people can easily find information on the internet, they are susceptible to easily accessible misinformation and fake news, especially information related to COVID-19 and vaccinations (Roozenbeek et al., 2020). The believers of conspiracy theories naturally oppose normative views and challenge widely accepted knowledge, often simply because it was prescribed by the authorities (Sternisko et al., 2020). Thus, a transparent practice by the government would certainly be able to boost people’s confidence, along with the support from scientific evidence.

Furthermore, the media is equally responsible for ensuring the dissemination of transparent and truthful (rather than sensational) public health information to the community. Overall, we predict that an increase in public awareness, knowledge, and understanding of the vaccines will further their uptake levels and that all of these outcomes are a product of transparent initiatives. Subsequently, the right actions and practices may be established to support the implementation of the vaccination program.

## Conclusion

Our results showed that anxiety related to COVID-19 has a positive effect on the attitudes toward vaccination. Secondly, we also revealed that belief in conspiracy theories has an inverse effect on vaccination attitudes. Subsequently, we also revealed that an individual’s resilience has a positive effect on the attitude toward vaccination and we found support on the moderating role of resilience to weaken the relationship between the inversed effect of conspiracy beliefs and attitudes toward vaccination. Besides, our results also demonstrated that though there are some similarities among gender, there are indeed some significant differences between female and male groups with regards to some hypothesized relationships. This implies that both groups are not entirely homogenous. To the best of our knowledge, the present study is the first empirical endeavor to simultaneously explore and demonstrate the effects of COVID-19 related anxiety, conspiracy beliefs and individual’s resilience with their attitude toward COVID-19 vaccines, as well as to examine the differences between male and female groups, particularly among Muslim people. In so doing, we have significantly contributed to the literature.

### Limitations and future directions

This study focused on the negative factors that affect people’s attitudes toward COVID-19 vaccines. Since one’s ability to withstand the negative forces might vary from one individual to another, future studies should therefore consider exploring other cognitive variables which may also contribute to enhancing vaccine acceptance. Moreover, this study was limited in that it focuses only on the Malaysian–Muslim context. Therefore, future studies could consider focusing on non-Muslim people, and alternatively compare Muslims and non-Muslims, as situations may have been different depending on religious interplays. We would also suggest that future studies consider using a qualitative design in conjunction with quantitative methods to gain deeper perspectives and insights into the various factors (and the relationships between them) that may affect peoples’ behavior toward COVID-19 vaccines.

## Data availability statement

The original contributions presented in this study are included in the article/supplementary material, further inquiries can be directed to the corresponding author.

## Ethics statement

Ethical review and approval was not required for the study on human participants in accordance with the local legislation and institutional requirements. The patients/participants provided their written informed consent to participate in this study.

## Author contributions

NaR, EJ, NuR, and NA contributed to design of the study and edited the manuscript. NaR organized the database, performed the statistical analysis, and wrote the original draft of the manuscript. All authors contributed to the manuscript revision, read, and approved the submitted version.
